# Combined Exposure to 1,2-Dichloropropane and Dichloromethane Enhances Hepatocellular Tumor Development in Mice

**DOI:** 10.3390/toxics14080676

**Published:** 2026-07-30

**Authors:** Min Gi, Masaki Fujioka, Arpamas Vachiraarunwong, Satoko Kawachi, Guiyu Qiu, Runjie Guo, Yurina Kawamura, Juncheng Pan, Yukina Kusunoki, Anna Kakehashi, Shugo Suzuki, Hideki Wanibuchi

**Affiliations:** 1 Department of Environmental Risk Assessment, Graduate School of Medicine, Osaka Metropolitan University, Osaka 545-8585, Japan; m22438g@omu.ac.jp (A.V.); si22394d@st.omu.ac.jp (G.Q.); sy23105k@st.omu.ac.jp (R.G.); st25261t@st.omu.ac.jp (Y.K.); su26581s@st.omu.ac.jp (J.P.); wani@omu.ac.jp (H.W.); 2 Department of Molecular Pathology, Graduate School of Medicine, Osaka Metropolitan University, Osaka 545-8585, Japan; b21405o@omu.ac.jp (M.F.); h21213r@omu.ac.jp (Y.K.); anna-k@omu.ac.jp (A.K.); shugo@omu.ac.jp (S.S.)

**Keywords:** 1,2-DCP, DCM, combined exposure, mice, hepatocarcinogenicity, gene expression alterations, tumor-associated pathways, occupational cholangiocarcinoma

## Abstract

Occupational cholangiocarcinoma among printing workers in Japan has raised concern regarding the carcinogenic hazards of chlorinated organic solvents, particularly 1,2-dichloropropane (1,2-DCP) and dichloromethane (DCM). Because workers were often exposed to multiple solvents, this study examined whether DCM modifies 1,2-DCP-associated hepatocellular tumor development in mice. Male C3H/HeN mice were administered a corn oil vehicle, 1,2-DCP alone at 500 mg/kg bw, or 1,2-DCP and DCM at 500 mg/kg bw each by oral gavage twice weekly for 52 weeks. Combined exposure significantly increased the incidence of hepatocellular adenomas (HCAs) compared with both the vehicle control and 1,2-DCP-alone groups. Tumor multiplicity was also significantly higher in the 1,2-DCP + DCM group than in the 1,2-DCP-alone group. HCAs from the 1,2-DCP + DCM group showed increased cell proliferative activity, 29 uniquely altered differentially expressed genes, significant upregulation of Gpc3 and Igfbp1, and downregulation of Dcn, consistent with altered tumor-associated molecular features. Canonical pathway analysis indicated suppression of xenobiotic metabolism, bile acid metabolism, and peroxisomal function in treatment-associated HCAs. These findings indicate that DCM co-exposure enhanced 1,2-DCP-associated hepatocellular tumor development and altered tumor-associated molecular features in mice, highlighting the importance of considering combined solvent exposure in chemical carcinogenic risk assessment.

## 1. Introduction

Occupational cholangiocarcinoma reported among workers in printing plants in Japan represents a serious example of chemically induced carcinogenesis associated with high-level exposure to chlorinated organic solvents. Epidemiological and clinical investigations identified a marked increase in cholangiocarcinoma among workers engaged in offset color proof-printing, where ink-cleaning agents and paint strippers containing 1,2-dichloropropane (1,2-DCP) and/or dichloromethane (DCM) had been used under conditions of poor ventilation and substantial vapor exposure [1,2]. Additional case and risk analyses have also supported the occupational association [3,4]. This incident and its epidemiological features have been summarized in a recent occupational-health mini-review [5]. In addition, molecular analysis of occupational cholangiocarcinoma has revealed distinctive mutational features associated with exposure to haloalkanes [6,7]. These findings raised major concerns regarding the carcinogenic hazards of volatile haloalkanes in occupational settings.

In response to the epidemiological evidence from Japan, the International Agency for Research on Cancer evaluated these compounds in Monograph Volume 110 [8]. IARC classified 1,2-DCP as Group 1, carcinogenic to humans, based on sufficient evidence for cholangiocarcinoma in humans and sufficient evidence of carcinogenicity in experimental animals. DCM was classified as Group 2A, probably carcinogenic to humans, based on sufficient evidence of carcinogenicity in experimental animals, limited evidence in humans, and strong mechanistic evidence involving GSTT1-mediated metabolism to reactive metabolites [8]. These classifications underscore the importance of clarifying the carcinogenic effects of these solvents, particularly under exposure conditions relevant to occupational settings.

1,2-DCP has been used as an intermediate in the production of chlorinated organic compounds, including tetrachloroethylene, trichloroethylene, and carbon tetrachloride, and has also been used as a metal-degreasing solvent and an activator in petroleum refining processes [8]. Experimental studies have demonstrated that 1,2-DCP induces nasal tumors in rats after long-term inhalation exposure [9] and hepatocellular adenomas and carcinomas in mice following long-term oral gavage exposure [10]. In vitro and in vivo studies have shown that 1,2-DCP exhibits genotoxic activity, with reported responses varying by experimental system, exposure condition, and species [11,12]. DCM has also been shown to induce hepatocellular and lung tumors in mice, as well as mammary gland tumors in rats, following long-term inhalation exposure [13,14]. In contrast to inhalation exposure, DCM did not show carcinogenicity in drinking-water studies in mice or rats [15]. Thus, although both chemicals possess carcinogenic potential, their effects appear to depend on species, target organ, and route of exposure.

A major challenge in interpreting the Japanese printing-plant cases is that workers were exposed to complex mixtures of chemicals rather than to a single agent. In addition to 1,2-DCP and DCM, other organic solvents, including 1,1,1-trichloroethane, petroleum-derived hydrocarbons, and surfactants, were reportedly used in the workplace [5,16]. Importantly, occupational cholangiocarcinoma cases in these workplaces often involved co-exposure to both 1,2-DCP and DCM, whereas exposure to either 1,2-DCP or DCM alone was less common [1,2]. Therefore, although epidemiological evidence strongly implicates 1,2-DCP as a principal causative agent, the possibility that co-exposure to DCM modified or enhanced the carcinogenic effects of 1,2-DCP remains an important issue for risk assessment [8].

Mechanistic evidence also suggests that these compounds may act through distinct but potentially interacting pathways. 1,2-DCP has shown genotoxic activity in several experimental systems and has been reported to cause hepatotoxicity and species-dependent alterations in hepatic xenobiotic-metabolizing enzymes [11,12]. DCM is metabolized through both oxidative and glutathione S-transferase-mediated pathways, and its carcinogenicity in mice has been associated with metabolic activation under inhalation exposure conditions [8]. Notably, previous experimental work demonstrated that combined inhalation exposure to 1,2-DCP and DCM enhanced genotoxicity in the liver of mice compared with exposure to either compound alone, supporting the possibility that concurrent exposure may enhance genotoxic or carcinogenic outcomes [12].

Although occupational cholangiocarcinoma is the key human health concern, experimental studies have shown that 1,2-DCP and DCM can induce tumors in rodents in a species-, target organ-, and exposure route-dependent manner. However, the carcinogenic effects of combined exposure to 1,2-DCP and DCM remain insufficiently understood. Because occupational cholangiocarcinoma cases in Japan occurred under mixed-solvent exposure conditions, experimental evaluation of concurrent exposure is essential for clarifying whether co-exposure enhances carcinogenic responses. Therefore, in the present study, we investigated the hepatocarcinogenic effects of repeated oral administration of 1,2-DCP alone and in combination with DCM in mice. We further examined proliferative activity, gene expression profiles, and pathway-level alterations in hepatocellular adenomas to clarify the biological features associated with combined exposure.

## 2. Materials and Methods

### 2.1. Chemicals

1,2-Dichloropropane (1,2-DCP; CAS No. 78-87-5; purity > 98%) and dichloromethane (DCM; CAS No. 75-09-2; purity > 99.5%) were purchased from Wako Pure Chemical Industries, Ltd. (Osaka, Japan). Corn oil was obtained from Nacalai Tesque, Inc. (Kyoto, Japan).

### 2.2. Animals and Husbandry

All animal experiments were conducted at the Animal Facility of Osaka City University Graduate School of Medicine and were approved by the Institutional Animal Care and Use Committee of Osaka City University Graduate School of Medicine, now Osaka Metropolitan University Graduate School of Medicine (#2014AH). The experiments were conducted in accordance with the Guidelines for Proper Conduct of Animal Experiments established by the Science Council of Japan in 2006.

A total of 65 male C3H/HeN mice, 7 weeks of age, were obtained from CLEA Japan, Inc. (Tokyo, Japan). The animals were housed in polycarbonate cages, with five mice per cage, in an experimental animal facility maintained at a target temperature of 22 ± 3 °C, relative humidity of 55 ± 5%, and a 12 h light/dark cycle. The animals were provided with a CE-2 basal pellet diet (CLEA Japan, Inc., Tokyo, Japan) and tap water ad libitum throughout the study. All animals were acclimatized to the animal room environment for 3 weeks before the start of the experiment.

### 2.3. Experimental Design

At 10 weeks of age, the mice were divided into three groups: vehicle control, 1,2-DCP-alone, and 1,2-DCP + DCM groups. Mice were administered corn oil vehicle, 1,2-DCP alone at 500 mg/kg body weight, or 1,2-DCP at 500 mg/kg body weight together with DCM at 500 mg/kg body weight by oral gavage twice weekly at 3-day intervals for 52 weeks. Test chemicals were dissolved in corn oil and administered at a volume of 5 mL/kg body weight. Body weight was measured immediately before each administration, and the gavage volume was adjusted accordingly. Food and water consumption were measured weekly. Preparation of the 1,2-DCP and DCM dosing solutions and oral gavage administration were performed in a draft chamber to minimize exposure to volatile solvents.

The dose of 1,2-DCP was selected based on a previous short-term hepatotoxicity study, in which oral administration of 500 mg/kg body weight 1,2-DCP for three consecutive days induced centrilobular hepatocellular necrosis in male B6C3F1 mice without apparent general toxicity [11]. This dose is higher than the hepatocarcinogenic dose of 250 mg/kg body weight used in the 2-year gavage carcinogenicity study in male B6C3F1 mice conducted by the National Toxicology Program [10].

A DCM-alone group was not included in the present study because DCM did not show carcinogenicity in 2-year drinking-water studies in rats or mice [15]. In addition, in our preliminary 4-week study, DCM administered at 500 mg/kg body weight by oral gavage five times per week caused no body-weight suppression, treatment-related hepatic histopathological changes, or induction of CYP2E1 or GSTT1 (unpublished data). Absolute and relative liver weights were slightly but significantly increased; however, these changes were not accompanied by hepatocellular necrosis or hypertrophy. This design also minimized the number of animals used in accordance with the principles of replacement, reduction, and refinement (3Rs) [17].

At the end of experimental week 52, mice were euthanized by inhalation of an overdose of isoflurane (Abbott Japan Co., Ltd., Tokyo, Japan) using a Small Animal Anesthetizer (MK-A110D; Muromachi Kikai Co., Ltd., Tokyo, Japan) coupled with an Anesthetic Gas Scavenging System (MK-T100E; Muromachi Kikai Co., Ltd., Tokyo, Japan). At necropsy, the livers were excised and weighed.

For comprehensive evaluation of macroscopic and microscopic liver lesions, all macroscopically visible lesions were sampled, and at least 10 liver sections were collected from each animal, including three sections from the left lateral lobe, three sections from the right middle lobe, three sections from the right lateral lobe, and one or more sections from the caudate lobe. The tissues were fixed in phosphate-buffered formalin, embedded in paraffin, and processed for hematoxylin and eosin staining and immunohistochemical analysis.

### 2.4. Immunohistochemistry

Hepatocellular adenomas (HCAs) available for analysis were examined immunohistochemically for Ki-67, a widely used marker of cellular proliferation, using the avidin–biotin–peroxidase complex method. HCAs were observed in 3, 3, and 14 mice from the vehicle control, 1,2-DCP-alone, and 1,2-DCP + DCM groups, respectively. Because two mice in the 1,2-DCP + DCM group harbored two HCAs each, a total of 3, 3, and 16 HCAs from the vehicle control, 1,2-DCP-alone, and 1,2-DCP + DCM groups, respectively, were analyzed for Ki-67 expression. For subsequent analyses, spontaneous HCAs in the vehicle control group were designated as C-HCAs, HCAs in the 1,2-DCP-alone group as D-HCAs, and HCAs in the 1,2-DCP + DCM group as DD-HCAs.

Briefly, liver sections containing HCAs were cut from paraffin-embedded liver specimens at 4 µm thickness, deparaffinized in xylene, and rehydrated through graded ethanol. Antigen retrieval was performed by microwaving at 98 °C for 20 min in 0.01 M citrate buffer (pH 6.0). Endogenous peroxidase activity was blocked with 0.3% H_2_O_2_ in distilled water for 5 min, and non-specific binding was blocked with normal goat serum at 37 °C for 30 min. Sections were then incubated with rabbit monoclonal anti-Ki-67 antibody [SP6] (ab16667; Abcam, Cambridge, MA, USA) at a dilution of 1:500 overnight at 4 °C. Immunoreactivity was detected using a VECTASTAIN Elite ABC Kit (Rabbit IgG) (PK-6101; Vector Laboratories, Burlingame, CA, USA) and 3,3′-diaminobenzidine hydrochloride (Sigma Chemical Co., St. Louis, MO, USA). Omission of the primary antibody served as the negative control and was included with each staining procedure.

### 2.5. RNA Extraction

Three C-HCAs from three mice in the vehicle control group, three D-HCAs from three mice in the 1,2-DCP-alone group, and six DD-HCAs from six mice in the 1,2-DCP + DCM group were processed for microarray analysis. These samples contained sufficient tumor tissue for RNA extraction and subsequent gene expression analysis.

Ten serial liver tumor sections were cut from paraffin-embedded liver specimens at 10 µm thickness. The first and last sections from each sample were stained with hematoxylin and eosin to identify the area for needle microdissection. After deparaffinization, tumor tissues and histologically normal liver tissues adjacent to the tumors were collected using sterile toothpicks under a light microscope and immediately transferred to Eppendorf tubes containing lysis buffer from the ReliaPrep FFPE Total RNA Miniprep System (Promega Co., Madison, WI, USA). Total RNA was extracted using the ReliaPrep FFPE Total RNA Miniprep System according to the manufacturer’s instructions.

### 2.6. Microarray Analysis

Microarray gene expression analysis was performed using the GeneChip Mouse Gene 2.0 ST Array (Affymetrix, Santa Clara, CA, USA) by Cell Innovator Inc. (Fukuoka, Japan). Raw data were processed using Affymetrix Expression Console 1.1 software. Signal intensity values were normalized using the SST-RMA method combined with the quantile normalization algorithm. Low-intensity signals with fluorescence values below 100 were excluded during the data-cleansing step.

Differentially expressed genes (DEGs) were defined as genes showing a z-score ≥ 2 and a fold change ≥ 2 for upregulation, or a z-score ≤ −2 and a fold change ≤ 0.5 for downregulation when compared with the corresponding non-tumorous liver tissues. DEG analysis was performed using Ingenuity Pathway Analysis (IPA; QIAGEN Build 9.0; QIAGEN Inc., Redwood City, CA, USA) with the Knowledge Base (Content Version 159584291; released 13 February 2026). Significant associations between DEGs and canonical pathways were determined using a right-tailed Fisher’s exact test, with significance set at *p* < 0.05. Pathway activation or inhibition was predicted using the IPA z-score algorithm, where a z-score ≥ 2 indicates predicted activation and a z-score ≤ −2 indicates predicted inhibition [18,19,20].

### 2.7. Quantitative Real-Time PCR

cDNA was synthesized from total RNA using SuperScript IV VILO Master Mix (Thermo Fisher Scientific K.K., Tokyo, Japan). Gene-specific primers and probes from TaqMan Gene Expression Assays were purchased from Thermo Fisher Scientific (Waltham, MA, USA). Quantitative real-time PCR (qPCR) was performed using TaqMan Fast Universal PCR Master Mix (Applied Biosystems, Foster City, CA, USA) and the Applied Biosystems 7500 Fast Real-Time PCR System (Applied Biosystems). Expression values for target genes were normalized to those of the housekeeping gene β2-microglobulin (B2m) using the comparative Ct method.

### 2.8. Statistical Analysis

All data are presented as the mean ± standard deviation (SD). Statistical analyses were performed using GraphPad Prism 11 (GraphPad Software, Inc., Boston, MA, USA). For continuous variables, equality of variances among groups was assessed using Bartlett’s test or the Brown–Forsythe test, as appropriate. When equal variances could be assumed, differences among the vehicle control, 1,2-DCP-alone, and 1,2-DCP + DCM groups were analyzed by ordinary one-way ANOVA followed by Tukey’s multiple-comparison test. When equal variances could not be assumed, Welch’s ANOVA followed by Dunnett’s T3 multiple-comparison test was used. For the Ki-67 analysis, the mouse was used as the experimental unit. Two mice each harbored two DD-HCAs; for these mice, the Ki-67 labeling indices of the two HCAs were averaged before statistical analysis. Because equal variances could not be assumed, the Ki-67 labeling indices were analyzed using Welch’s ANOVA followed by Dunnett’s T3 multiple-comparison test. Tumor incidence was evaluated using a two-sided Fisher’s exact test. Tumor multiplicity, defined as the number of tumors per mouse, was analyzed using the Kruskal–Wallis test followed by Dunn’s multiple-comparison test because the data were discrete and not normally distributed. A value of *p* < 0.05 was considered statistically significant.

## 3. Results

### 3.1. General Observations

Survival, final body and liver weights, and food and water consumption are summarized in Table 1. By week 52, one mouse in the vehicle control group and four mice in the 1,2-DCP-alone group had died, leaving 14, 21, and 25 surviving mice in the vehicle control, 1,2-DCP-alone, and 1,2-DCP + DCM groups, respectively.

Final body weights and absolute liver weights did not differ significantly among the groups. Relative liver weights were significantly increased in both the 1,2-DCP-alone and 1,2-DCP + DCM groups compared with the vehicle control group. Average food and water consumption were slightly lower in the treated groups than in the vehicle control group.

### 3.2. HCA Development in the Liver

Histopathological findings in the liver are summarized in Table 2. HCAs were observed in all groups and were considered spontaneous in the vehicle control group. One mouse in the 1,2-DCP-alone group that died at week 15 had an HCA; therefore, mice that survived until week 15 or later were included in the histopathological evaluation.

The incidence of HCAs was significantly higher in the 1,2-DCP + DCM group (14/25, 56.0%) than in the vehicle control group (3/14, 21.4%) and the 1,2-DCP-alone group (4/23, 17.4%). Tumor multiplicity was significantly higher in the 1,2-DCP + DCM group than in the 1,2-DCP-alone group. Although tumor multiplicity was also higher than in the vehicle control group, the difference was not statistically significant. No histopathological alterations were observed in the bile ducts in any group.

### 3.3. Proliferative Activity of HCAs

**Figure 1:**
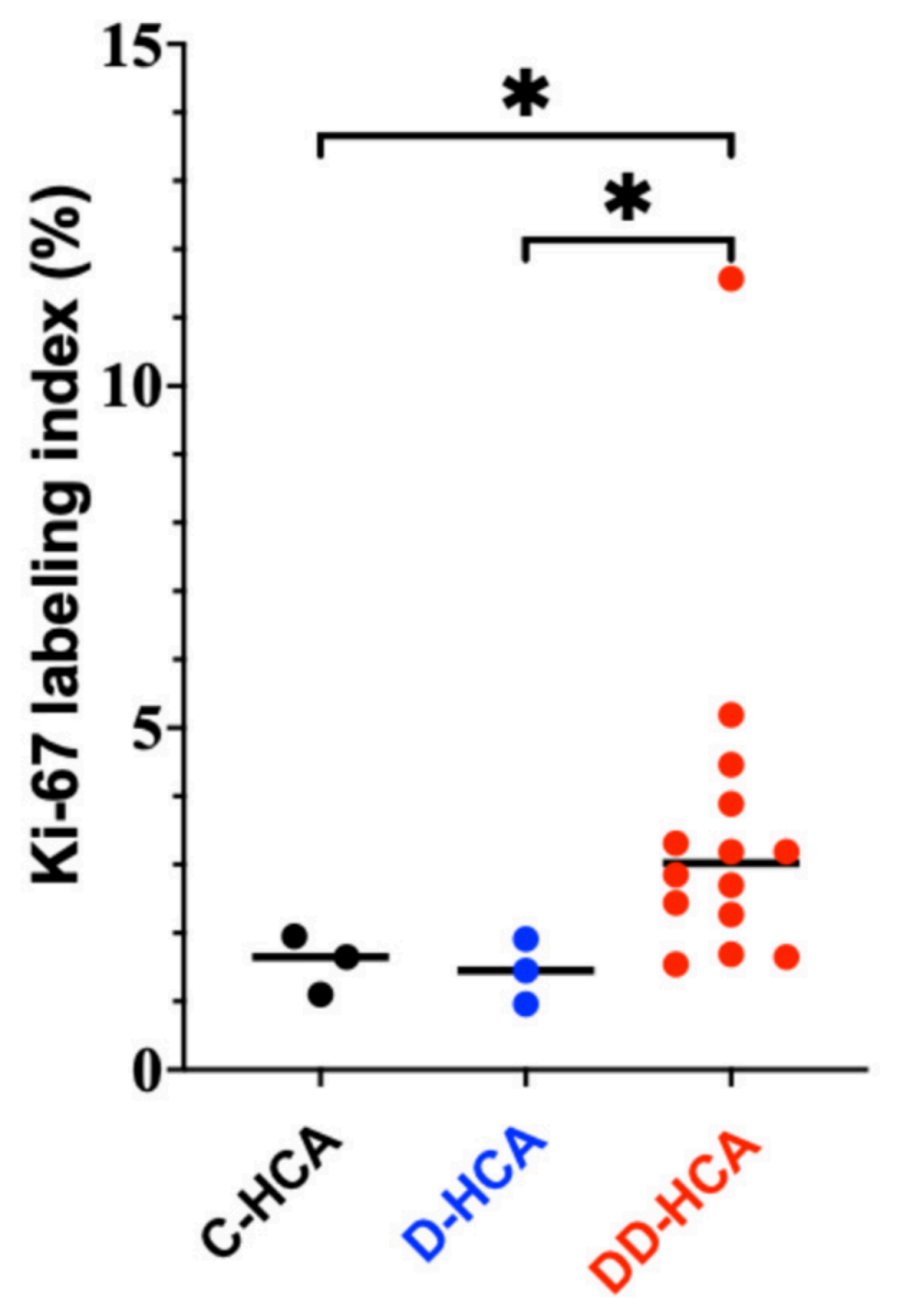
Proliferative activity of HCAs. Ki-67 labeling indices in HCAs from the vehicle control, 1,2-DCP-alone, and 1,2-DCP + DCM groups are shown. The Ki-67 labeling index was significantly higher in DD-HCAs than in C-HCAs and D-HCAs. C-HCA, spontaneous HCA in the vehicle control group; D-HCA, HCA in the 1,2-DCP-alone group; DD-HCA, HCA in the 1,2-DCP + DCM group. Each dot represents one mouse. Two mice each contributed two DD-HCAs, and the mean Ki-67 labeling index of the two tumors was used; all other mice contributed one HCA. Horizontal bars indicate the mean. * *p* < 0.05.

Ki-67 immunohistochemistry was performed to evaluate proliferative activity in HCAs. The Ki-67 labeling index was significantly higher in DD-HCAs than in C-HCAs and D-HCAs (Figure 1).

### 3.4. Distinct Gene Expression Profiles in HCAs

Gene expression profiles were compared between HCAs and the corresponding non-tumorous liver tissues in each group. In C-HCAs, 96 DEGs were identified, including 53 upregulated and 43 downregulated genes. In D-HCAs, 136 DEGs were identified, including 64 upregulated and 72 downregulated genes. In DD-HCAs, 128 DEGs were identified, including 47 upregulated and 81 downregulated genes.

**Figure 2:**
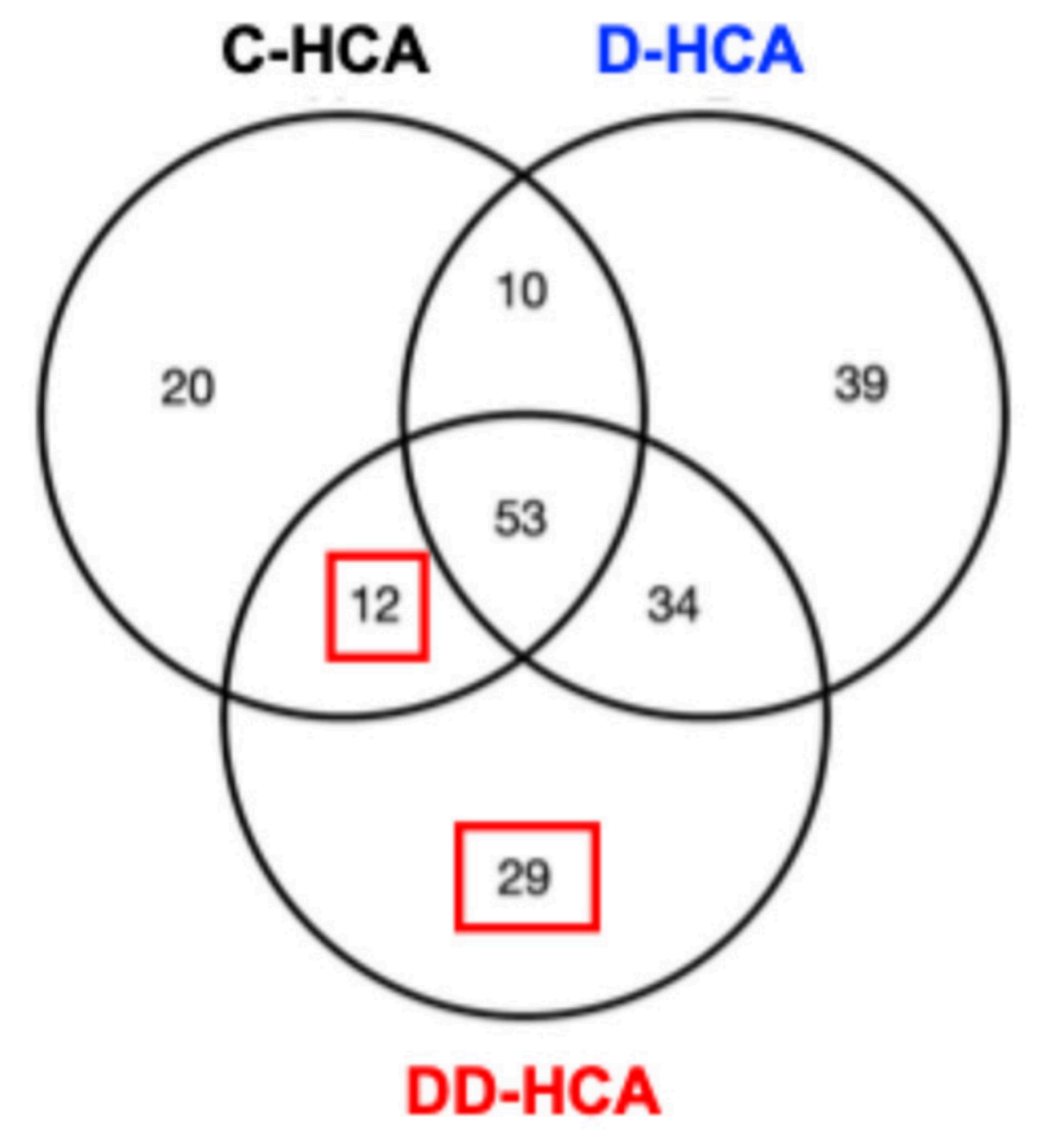
Numbers of DEGs identified in C-HCAs, D-HCAs, and DD-HCAs. A total of 53 DEGs were commonly altered in all three HCA groups, whereas 29 DEGs were uniquely altered in DD-HCAs. In addition, 41 DEGs were significantly altered in DD-HCAs but not in D-HCAs, including the 29 DD-HCA-specific DEGs and 12 additional DEGs that were altered in both DD-HCAs and C-HCAs but not in D-HCAs. C-HCA, spontaneous HCA in the vehicle control group; D-HCA, HCA in the 1,2-DCP-alone group; DD-HCA, HCA in the 1,2-DCP + DCM group.

Venn diagram analysis showed that 53 DEGs were common to all three HCA groups, 34 were shared only between D-HCAs and DD-HCAs, and 29 were uniquely altered in DD-HCAs (Figure 2, Table 3).

Among the 29 DEGs uniquely altered in DD-HCAs, *Gpc3* was upregulated, whereas genes associated with bile acid transport (*Abcb11*, *Nr1h4*, and *Slc27a5*), xenobiotic metabolism (*Cyp2d9*, *Cyp4f15*, *Gsta3*, *Ugt2b34*, and *Alas1*), lipid metabolism (*Acadsb*, *Acsm1*, *Pemt*, and *Thrsp*), amino acid/one-carbon metabolism (*Asl* and *Gnmt*), and extracellular matrix regulation (*Dcn*) were downregulated.

To further compare D-HCAs and DD-HCAs, genes significantly altered in DD-HCAs but not in D-HCAs were examined. This analysis identified 41 DEGs (Table S1), comprising the 29 DD-HCA-specific DEGs and 12 additional DEGs altered in both DD-HCAs and C-HCAs but not in D-HCAs (Figure 2). This set included *Igfbp1* and genes associated with tumor/growth signaling, bile acid transport, xenobiotic metabolism, lipid metabolism, amino acid/one-carbon metabolism, immune/inflammatory regulation, and extracellular matrix regulation.

### 3.5. Validation of Tumor-Associated and Growth-Regulatory Genes

qPCR was performed for *Gpc3*, *Igfbp1*, and *Dcn*, which were altered in DD-HCAs but not in D-HCAs and were selected to represent tumor-associated signaling, growth-factor regulation, and extracellular matrix regulation, respectively.

**Figure 3:**
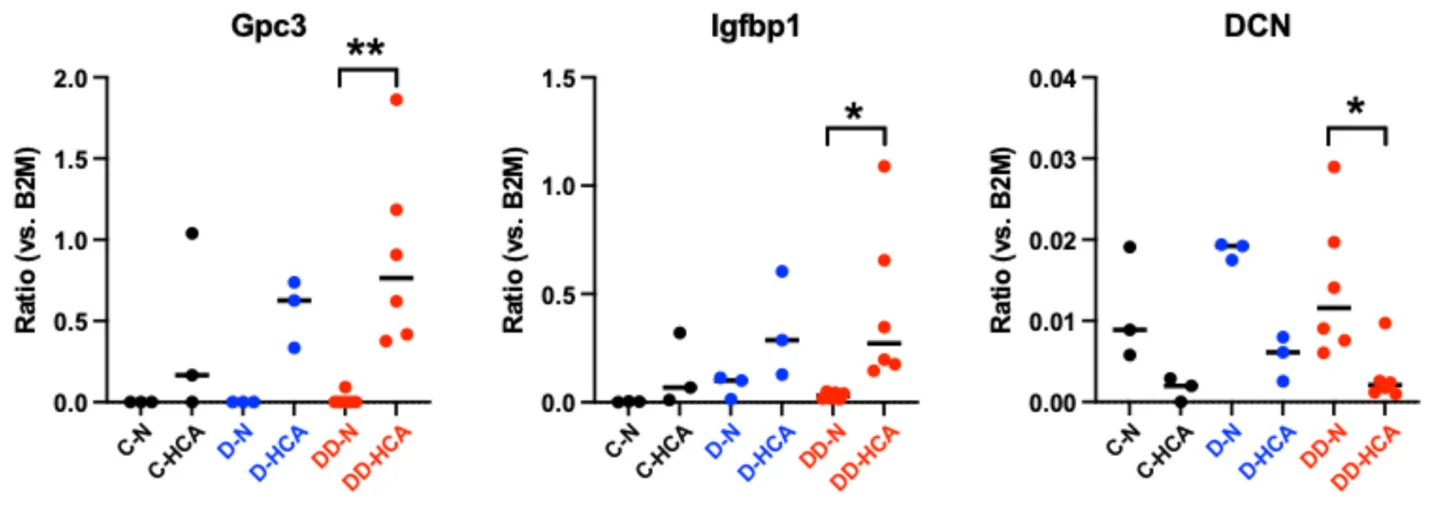
Relative mRNA expression levels of *Gpc3*, *Igfbp1*, and *Dcn* in HCAs and corresponding non-tumorous liver tissues. *Gpc3* and *Igfbp1* were significantly upregulated in DD-HCAs compared with corresponding non-tumorous liver tissues (DD-N), whereas *Dcn* was significantly downregulated in DD-HCAs. No statistically significant changes were observed in D-HCAs or C-HCAs compared with their corresponding non-tumorous liver tissues. C-N, D-N, and DD-N indicate non-tumorous liver tissues corresponding to C-HCAs, D-HCAs, and DD-HCAs, respectively. Each dot represents one HCA or non-tumorous liver tissue, and horizontal bars indicate the mean. * *p* < 0.05, ** *p* < 0.01.

*Gpc3* and *Igfbp1* were significantly upregulated in DD-HCAs compared with the corresponding non-tumorous liver tissues, whereas *Dcn* was significantly downregulated (Figure 3). No statistically significant changes were observed in D-HCAs compared with their corresponding non-tumorous tissues.

### 3.6. Canonical Pathway Alterations in HCAs

**Figure 4:**
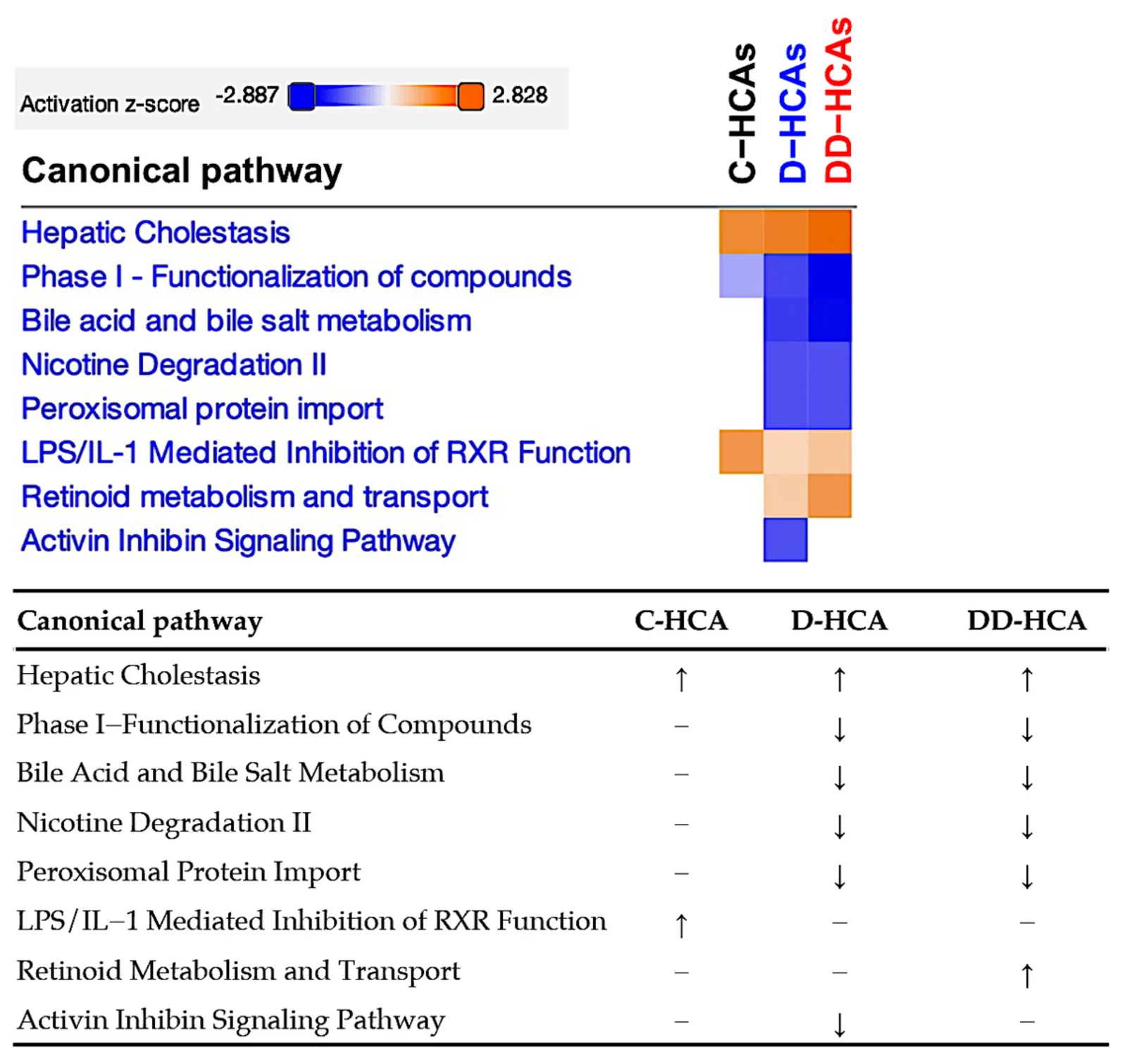
Canonical pathway alterations in HCAs. The heatmap shows IPA-predicted activation z-scores for canonical pathways that were predicted to be activated or inhibited in at least one HCA group. The accompanying table summarizes the predicted activation status of each pathway. ↑, predicted activation; ↓, predicted inhibition; –, not predicted to be significantly activated or inhibited based on the IPA activation z-score criteria. C-HCA, spontaneous HCA in the vehicle control group; D-HCA, HCA in the 1,2-DCP-alone group; DD-HCA, HCA in the 1,2-DCP + DCM group.

Canonical pathway analysis using IPA was performed to compare predicted pathway alterations among C-HCAs, D-HCAs, and DD-HCAs (Figure 4). Hepatic Cholestasis was predicted to be activated in all three HCA groups. Phase I—Functionalization of Compounds, Bile Acid and Bile Salt Metabolism, Nicotine Degradation II, and Peroxisomal Protein Import were predicted to be inhibited in both D-HCAs and DD-HCAs but not in C-HCAs. LPS/IL-1-Mediated Inhibition of RXR Function was predicted to be activated only in C-HCAs; Retinoid Metabolism and Transport, only in DD-HCAs; and Activin–Inhibin Signaling Pathway was predicted to be inhibited only in D-HCAs.

## 4. Discussion

The present study evaluated the hepatocarcinogenic potential of combined exposure to 1,2-DCP and DCM using a 52-week repeated oral administration model in mice. Combined exposure significantly increased the incidence of HCAs compared with both the vehicle control and 1,2-DCP-alone groups and increased tumor multiplicity compared with the 1,2-DCP-alone group. In contrast, 1,2-DCP alone did not significantly increase HCA incidence or multiplicity under the present experimental conditions. These findings indicate that co-exposure to 1,2-DCP and DCM enhanced hepatocellular tumor development compared with exposure to 1,2-DCP alone in this model.

The occurrence of spontaneous HCAs in the vehicle control group is consistent with the known background development of hepatocellular tumors in mice [21,22]. Although 1,2-DCP alone has been reported to induce hepatocellular tumors in a 2-year gavage carcinogenicity study [10], the absence of a significant increase in the present study may be related to the shorter experimental duration and twice-weekly administration schedule. This experimental setting nevertheless provided an appropriate model for examining whether DCM co-exposure modifies 1,2-DCP-associated hepatocellular tumor development.

At the molecular level, DD-HCAs showed alterations in tumor-associated and growth-regulatory genes together with broad suppression of genes involved in differentiated hepatic functions, including bile acid transport, xenobiotic metabolism, lipid metabolism, amino acid/one-carbon metabolism, and extracellular matrix regulation (Table 3 and Table S1). Among the genes altered in DD-HCAs, *Gpc3*, *Igfbp1*, and *Dcn* were of particular interest because of their reported roles in hepatocellular tumor biology, growth-factor signaling, and extracellular matrix regulation.

*Gpc3* encodes glypican-3, a cell-surface heparan sulfate proteoglycan that is frequently overexpressed in hepatocellular carcinoma and is recognized as a diagnostic and therapeutic target in liver cancer [23]. GPC3 has also been implicated in the modulation of Wnt/β–catenin and other growth-related signaling pathways [24]. Therefore, the upregulation of *Gpc3* in DD-HCAs is consistent with a hepatocellular tumor-associated and growth-related phenotype.

*Igfbp1* encodes insulin-like growth factor-binding protein 1, a hepatocyte-derived member of the IGF-binding protein family that regulates IGF bioavailability and IGF-related signaling. IGFBP1 has shown context-dependent effects in hepatocellular carcinoma, including reported anti-invasive activity [25]. Dysregulation of the IGF/IGF-1R axis has been implicated in tumor growth, stemness-related properties, and therapeutic resistance [26]. IGFBP1 has also been associated with tumor migration, invasion, growth, and resistance to tyrosine kinase inhibitors [27,28]. Thus, its upregulation in DD-HCAs is consistent with altered IGF-related signaling under combined-exposure conditions.

In contrast, *Dcn* encodes decorin, a small leucine-rich extracellular matrix proteoglycan involved in tissue architecture and growth-factor regulation. Decorin has been reported to exert tumor-suppressive effects in hepatocarcinogenesis through regulation of receptor tyrosine kinase signaling and suppression of liver tumor development [29,30]. It is also recognized as an extracellular matrix-associated oncosuppressive proteoglycan that modulates tumor growth, angiogenesis, and cancer-related signaling pathways [31]. Therefore, the downregulation of *Dcn* in DD-HCAs may reflect the loss of an extracellular matrix-associated tumor-suppressive influence.

Taken together, these gene expression changes are consistent with recognized molecular features of hepatocellular tumors, in which activation of growth-related signaling is accompanied by loss of mature hepatocyte differentiation and metabolic functions [32,33,34]. Molecular classification studies of human hepatocellular carcinoma have identified subclasses characterized by proliferation-associated signaling, Wnt/β–catenin-related features, and differences in hepatocyte differentiation status [35,36]. Maintenance of hepatocyte identity is closely linked to liver-specific metabolic functions [37], whereas malignant transformation is often accompanied by remodeling or suppression of xenobiotic metabolism and bile acid-related pathways [38,39]. Consistent with these findings, canonical pathway analysis predicted inhibition of pathways related to xenobiotic metabolism, bile acid metabolism, and peroxisomal function, particularly in D-HCAs and DD-HCAs (Figure 4). Thus, the altered tumor-associated gene expression and suppression of hepatic metabolic pathways in DD-HCAs support a more tumor-associated hepatocellular phenotype.

The present findings are relevant to the assessment of combined solvent exposure. Occupational cholangiocarcinoma among printing workers exposed to chlorinated organic solvents raised concern regarding the carcinogenic hazards of 1,2-DCP and DCM [5,8]. Although the present study focused on hepatocellular rather than biliary tumors, species- and organ-specific differences in tumor development are common in chemical carcinogenesis [40,41]. The comparison between the 1,2-DCP-alone and 1,2-DCP + DCM groups provides experimental evidence that the addition of DCM can enhance hepatocellular tumor development under concurrent exposure conditions. This finding is consistent with previous evidence that combined inhalation exposure to 1,2-DCP and DCM enhanced genotoxicity in mouse liver compared with exposure to either compound alone [12]. These results support the importance of evaluating combined chemical exposure, rather than single-agent exposure alone, in carcinogenic hazard assessment.

An important limitation of the present study is the absence of a DCM-alone group. Therefore, although combined exposure increased hepatocellular tumor development compared with 1,2-DCP alone, it remains unclear whether this increase reflects an independent effect of DCM, an additive effect, or an interaction between the two chemicals. Gene expression analyses were performed using FFPE tumor samples, and the transcriptomic findings should be considered exploratory because the number of tumors available for analysis was limited and fixation-related effects may have influenced the results.

It should also be noted that the exposure conditions used in the present study differed from those experienced by printing workers. Occupational exposure occurred predominantly through inhalation [1,5,16], whereas the mice received relatively high doses by repeated oral gavage to ensure controlled and reproducible administration. Because the exposure route can influence toxicokinetics and target-organ responses [8], the present findings should not be directly extrapolated quantitatively to occupational exposure scenarios. Rather, they provide experimental hazard information on the effects of concurrent exposure to 1,2-DCP and DCM.

## 5. Conclusions

The present study demonstrates that combined exposure to 1,2-DCP and DCM enhanced hepatocellular tumor development and tumor cell proliferative activity in mice compared with exposure to 1,2-DCP alone. DD-HCAs exhibited distinct molecular features, including altered tumor-associated gene expression and suppression of differentiated hepatic metabolic functions. These findings provide experimental hazard information relevant to the combined solvent exposures implicated in occupational cholangiocarcinoma and highlight the importance of considering chemical mixtures in carcinogenic hazard assessment.

## Figures and Tables

**Table 1:** Final body and liver weights and food and water consumption in mice.

Group	Initial No. of Mice	Final No. of Mice	Final Body Weight (g)	Absolute Liver Weight (g)	Relative Liver Weight (%)	Average Food Consumption (g/Day/Mouse)	Average Water Consumption (g/Day/Mouse)
Control (Vehicle)	15	14	29.0 ± 1.3	1.4 ± 0.1	4.7 ± 0.3	3.5 ± 0.4	5.1 ± 1.1
1,2-DCP	25	21	28.8 ± 1.1	1.4 ± 0.1	5.0 ± 0.4 **	3.3 ± 0.3	4.5 ± 0.6
1,2-DCP + DCM	25	25	28.3 ± 1.8	1.5 ± 0.4	5.2 ± 1.3 **	3.2 ± 0.2	4.4 ± 0.4

** *p* < 0.01 vs. control (vehicle).

**Table 2:** Incidence and multiplicity of HCAs in mice.

Group	Effective No. of Mice Examined ^a^	Incidence of HCA (%)	Multiplicity of HCA (No./Mouse)
Control (Vehicle)	14	3/14 (21.4%)	0.2 ± 0.4
1,2-DCP	23	4/23 (17.4%)	0.2 ± 0.4
1,2-DCP + DCM	25	14/25 (56.0%) * ††	0.6 ± 0.6 †

^a^ Effective number of mice examined for hepatocellular adenoma (HCA), including mice that survived until week 15 or later, because one HCA was observed in a mouse that died at week 15 in the 1,2-DCP group. * *p* < 0.05 vs. control (vehicle); † *p* < 0.05 vs. 1,2-DCP; †† *p* < 0.01 vs. 1,2-DCP.

**Table 3:** Twenty-nine DEGs uniquely altered in DD-HCAs.

		Fold Changes of DEGs (vs. the Corresponding Non-Tumorous Liver Tissues)			
Symbol	Gene Name	C-HCA *	D-HCA *	DD-HCA *	Function Classification
*Gpc3*	glypican 3	–	–	7.6	Tumor/growth signaling
*Ighj3*	immunoglobulin heavy joining 3	–	–	3.4	Immune-related
*Apom*	apolipoprotein M	–	–	2.8	Lipid metabolism
*Dusp6*	dual specificity phosphatase 6	–	–	2.7	MAPK/ERK signaling regulation
*Mir-188*	relatives of microRNA 188	–	–	2.3	Post-transcriptional regulation
*Abcb11*	ATP-binding cassette subfamily B member 11	–	–	−2.7	Bile acid transport
*Acadsb*	acyl-CoA dehydrogenase short/branched chain	–	–	−2.7	Lipid metabolism
*Pemt*	phosphatidylethanolamine N-methyltransferase	–	–	−2.7	Lipid metabolism
*Nr1h4*	nuclear receptor subfamily 1 group H member 4	–	–	−2.7	Bile acid transport
*Ldhd*	lactate dehydrogenase D	–	–	−2.8	Energy metabolism
*Slc27a5*	solute carrier family 27 member 5	–	–	−2.8	Bile acid transport
*Serpina11*	serine peptidase inhibitor, clade A, member 11	–	–	−2.8	Protease regulation
*Ugt2b34*	UDP glucuronosyltransferase 2 family, polypeptide B34	–	–	−2.9	Xenobiotic metabolism
*Asl*	argininosuccinate lyase	–	–	−3.1	Amino acid metabolism
*Mir1948*	microRNA 1948	–	–	−3.2	Post-transcriptional regulation
*Alas1*	5′-aminolevulinate synthase 1	–	–	−3.2	Xenobiotic metabolism
*Hsd17b11*	hydroxysteroid 17-beta dehydrogenase 11	–	–	−3.4	Lipid metabolism
*Thrsp*	thyroid hormone responsive	–	–	−3.5	Lipid metabolism
*Gsta3*	glutathione S-transferase alpha 3	–	–	−3.6	Xenobiotic metabolism
*Atp11c*	ATPase phospholipid transporting 11C	–	–	−3.8	Lipid/membrane regulation
*Dpyd*	dihydropyrimidine dehydrogenase	–	–	−3.9	Nucleotide metabolism
*Cyp2d9*	cytochrome P450 family 2 subfamily d polypeptide 9	–	–	−4	Xenobiotic metabolism
*Hamp2*	hepcidin antimicrobial peptide 2	–	–	−4.1	Iron homeostasis/immune-related
*Acsm1*	acyl-CoA synthetase medium-chain family member 1	–	–	−4.2	Lipid metabolism
*Dcn*	decorin	–	–	−4.2	Extracellular matrix regulation
*Ly6a*	lymphocyte antigen 6 family member A	–	–	−4.3	Immune/injury-response-related
*Gnmt*	glycine N-methyltransferase	–	–	−4.3	Amino acid metabolism
*Cyp4f15*	cytochrome P450 family 4 subfamily f polypeptide 15	–	–	−5.7	Xenobiotic metabolism
*Asic5*	acid-sensing ion channel subunit family member 5	–	–	−5.9	Others/unclear

* C-HCA, spontaneous HCA in the vehicle control group; D-HCA, HCA in the 1,2-DCP-alone group; DD-HCA, HCA in the 1,2-DCP + DCM group; –, gene did not meet the criteria for differentially expressed genes (DEGs).

## Data Availability

The raw data supporting the conclusions of this article will be made available by the authors upon request.

## Supplementary Materials

The following supporting information can be downloaded at https://www.mdpi.com/article/10.3390/toxics14080676/s1, Table S1: Forty-one DEGs significantly altered in DD-HCAs but not in D-HCAs.

## Abbreviations

1,2-DCP	1,2-dichloropropane
3Rs	replacement, reduction, and refinement
ABC	avidin-biotin–peroxidase complex
B2m	beta-2-microglobulin
CYP	cytochrome P450
DCM	dichloromethane
DEG	differentially expressed gene
FFPE	Formalin-fixed paraffin-embedded
GPC3	glypican-3
GSTT1	glutathione S-transferase theta 1
HCA	hepatocellular cell adenoma
IARC	International Agency for Research on Cancer
IGFBP1	insulin-like growth factor-binding protein 1
IPA	Ingenuity Pathway Analysis
