# Supplementary material for: Combined Exposure to 1,2-Dichloropropane and Dichloromethane Enhances Hepatocellular Tumor Development in Mice
Source: Toxics. 2026 Jul 30;14(8):676. doi: 10.3390/toxics14080676 (PMC13517227; doi:10.3390/toxics14080676)
Supplement: Supplementary file 1 [file toxics-14-00676-s001.zip › toxics-4435190-supplementary.pdf]

Table S1. Forty-one DEGs significantly altered in DD-HCAs but not in D-HCAs*.

Symbol	Gene Name	Fold changes of DEGs (vs. the corresponding non-tumorous liver tissues)			Function classification
		C-HCA	D-HCA	DD-HCA	
Tmem54	transmembrane protein 54	10.2	–	7.7	Membrane-associated/unclear
Gpc3	glypican 3	–	–	7.6	Tumor/growth signaling
Bex2	brain expressed X-linked 2	–	–	5.7	Tumor/growth signaling
Il1rn	interleukin 1 receptor antagonist	–	–	4.4	Inflammation/immune regulation
n-R5s1	nuclear encoded rRNA 5S 1	–	–	4.0	Others/unclear
Ighj3	immunoglobulin heavy joining 3	–	–	3.4	Immune-related
Igfbp1	insulin-like growth factor binding protein 1	–	–	3.2	Growth-factor signaling
Apom	apolipoprotein M	–	–	2.8	Lipid metabolism
Dusp6	dual specificity phosphatase 6	–	–	2.7	MAPK/ERK signaling regulation
Mir-188	relatives of microRNA 188	–	–	2.3	Post-transcriptional regulation
Gm15567	predicted gene 15567	–	–	2.2	Others/unclear
Gm22956	predicted gene 22956	3.0	–	2.1	Others/unclear
Abcb11	ATP binding cassette subfamily B member 11	–	–	-2.7	Bile acid transport
Acadsb	acyl-CoA dehydrogenase short/branched chain	–	–	-2.7	Lipid metabolism
Pemt	phosphatidylethanolamine N-methyltransferase	–	–	-2.7	Lipid metabolism
Nr1h4	nuclear receptor subfamily 1 group H member 4	–	–	-2.7	Bile acid transport
Ldhd	lactate dehydrogenase D	–	–	-2.8	Energy metabolism
Slc27a5	solute carrier family 27 member 5	–	–	-2.8	Bile acid transport
Serpina11	serine peptidase inhibitor, clade A, member 11	–	–	-2.8	Protease regulation
Ugt2b34	UDP glucuronosyltransferase 2 family, polypeptide B34	–	–	-2.9	Xenobiotic metabolism
Asl	argininosuccinate lyase	–	–	-3.1	Amino acid metabolism
Cyp2d13	cytochrome P450 family 2 subfamily d polypeptide 13	-3.1	–	-3.2	Xenobiotic metabolism
Mir1948	microRNA 1948	–	–	-3.2	Post-transcriptional regulation
Fmo1	flavin-containing dimethylaniline monooxygenase 1	–	–	-3.2	Xenobiotic metabolism
Alas1	5′-aminolevulinate synthase 1	–	–	-3.2	Xenobiotic metabolism
Ang	angiogenin	–	–	-3.4	Angiogenesis / growth regulation
Hsd17b11	hydroxysteroid 17-beta dehydrogenase 11	–	–	-3.4	Lipid metabolism
Thrsp	thyroid hormone responsive	–	–	-3.5	Lipid metabolism
Gsta3	glutathione S-transferase alpha 3	–	–	-3.6	Xenobiotic metabolism
Atp11c	ATPase phospholipid transporting 11C	–	–	-3.8	Lipid/membrane regulation
Lect2	leukocyte cell-derived chemotaxin 2	-2.1	–	-3.9	Immune/inflammatory regulation
Dpyd	dihydropyrimidine dehydrogenase	–	–	-3.9	Nucleotide metabolism
Cyp2d9	cytochrome P450 family 2 subfamily d polypeptide 9	–	–	-4.0	Xenobiotic metabolism
Hamp2	hepcidin antimicrobial peptide 2	–	–	-4.1	Iron homeostasis/immune-related
Hsd3b6	hydroxy-delta-5-steroid dehydrogenase, 3 beta- and steroid delta-isomerase 6	-2.9	–	-4.1	Steroid/lipid metabolism
Acsm1	acyl-CoA synthetase medium-chain family member 1	–	–	-4.2	Lipid metabolism
Dcn	decorin	–	–	-4.2	Extracellular matrix regulation
Ly6a	lymphocyte antigen 6 family member A	–	–	-4.3	Immune/injury-response related
Gnmt	glycine N-methyltransferase	–	–	-4.3	Amino acid metabolism
Cyp4f15	cytochrome P450 family 4 subfamily f polypeptide 15	–	–	-5.7	Xenobiotic metabolism
Asic5	acid-sensing ion channel subunit family member 5	–	–	-5.9	Others/unclear

*This table includes 41 DEGs that were significantly altered in DD-HCAs but not in D-HCAs. Among these genes, 29 were uniquely altered in DD-HCAs, whereas 12 additional DEGs were altered in both DD-HCAs and C-HCAs but not in D-HCAs. C-HCA, spontaneous HCA in the vehicle control group; D-HCA, HCA in the 1,2-DCP-alone group; DD-HCA, HCA in the 1,2-DCP + DCM group. –, gene did not meet the criteria for differentially expressed genes (DEGs).
